# Charge Transfer Quenching and Maximum of a Liquid–Air Contact Line Moving over a Hydrophobic Surface

**DOI:** 10.1021/acs.langmuir.3c03605

**Published:** 2024-02-14

**Authors:** Lars Egil Helseth

**Affiliations:** Department of Physics and Technology, University of Bergen, Allegaten 55, Bergen 5020, Norway

## Abstract

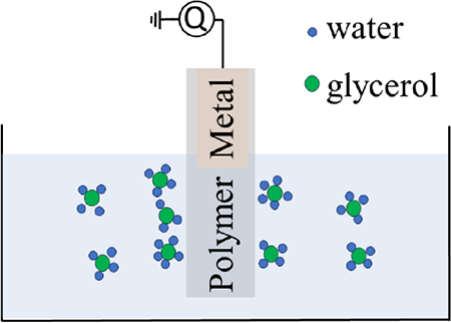

Charge transfer when a hydrophobic fluoropolymer surface comes in contact with salt solutions of water, methanol, and glycerol is investigated. It is found that the charge transfer decreases faster with an increasing fraction of glycerol in water than it does with methanol in water. It is also demonstrated that for both mixtures, the charge transfer increases with the amount of added sodium chloride for small concentrations but then reaches a maximum and subsequently decreases. Surprisingly, this maximum charge transfer shifts toward higher salt concentrations with increasing amount of glycerol in water. However, in water–methanol mixtures, one does not observe a similar shift in charge transfer maximum toward higher salt concentrations. These observations are explained using a model, taking into account the decreased shear distance from the hydrophobic surface for which ions are removed from the electrical double layer due to an interplay of forces acting on the ions.

## Introduction

Electric charge is transferred when water comes into contact with a solid or liquid surface.^1−4^ The surface charge state depends on both the surface and liquid compositions^4−6^ and is known to control the liquid spreading dynamics.^7^ Hydrophobic surfaces, for example fluoropolymers, usually acquire a net negative charge, such that a corresponding net positive charge remains in the liquid.^8−10^ The detailed origin of this charge transfer is not well understood, but investigations have suggested that hydroxide ions^11,12^ or alignment of the water molecules and topological defects in the hydrogen bonding structure near the interface^13^ are likely to play major roles. The arrangement of the solid surface itself, such as its roughness,^14^ and the initial charged state^15−20^ play important roles for the observed charge transfer due to passing water–air contact lines. However, also the composition of the liquid,^21−30^ the manner in which it impacts the solid surface,^3,31−34^ the flow,^35,36^ and the slide length^37,38^ influence the charge transfer. One may argue that a completely satisfactory picture of the charge transfer as the three-phase contact line moves past the solid has not been found, and this topic needs further investigation.

The surface charge and electrokinetic properties of liquids in contact with hydrophobic surfaces have been investigated extensively.^39−41^ However, the introduction of a gas phase appears to complicate the analysis and the contact charge transfer observed when a three-phase contact line moves over a fluoropolymer surface is not well understood. Several studies have revealed that the charge transfer may increase or decrease with ion concentration,^24−30,42^ and this has recently been explained to be due to a combination of shear, which removes charge in the electrical double layer^30^ with quenching of the active sites near the fluoropolymer.^29^ However, it is not clear how other aqueous liquids miscible with water influence the quenching when hydration takes place near the interface. Moreover, it is also not known how such mixing influences the charge removal from the electrical double layer. Experimental data are important if one is to better understand and improve the mechanisms behind the transfer of charge occurring in sensors and energy harvesting devices.

In the current work, these two questions are addressed and measurements of the charge transfer occurring when salt solutions of water, methanol, and glycerol come in contact with a hydrophobic fluoropolymer are reported. There are several reasons why methanol and glycerol are selected. Both are small and readily available molecules containing only methyl and hydroxyl groups, which can alter the hydrogen bonding network of water without being too sensitive to alignment as one would expect for larger hydrocarbon chains. Their interaction with bulk water is well studied but not their interaction with water molecules near hydrophobic surfaces. The physicochemical properties of glycerol and methanol and their mixtures with bulk water are available in the literature. This is particularly interesting here, since the aim is to compare how charge transfer changes in mixtures with water with or without added salt. The reported experimental data appear to be reasonably well explained by recent theories^29,30^ and allow extraction of parameters, which may lead to a better understanding of liquid–solid contact charge transfer mechanisms in liquids.

## Materials and Methods

**Figure 1:**
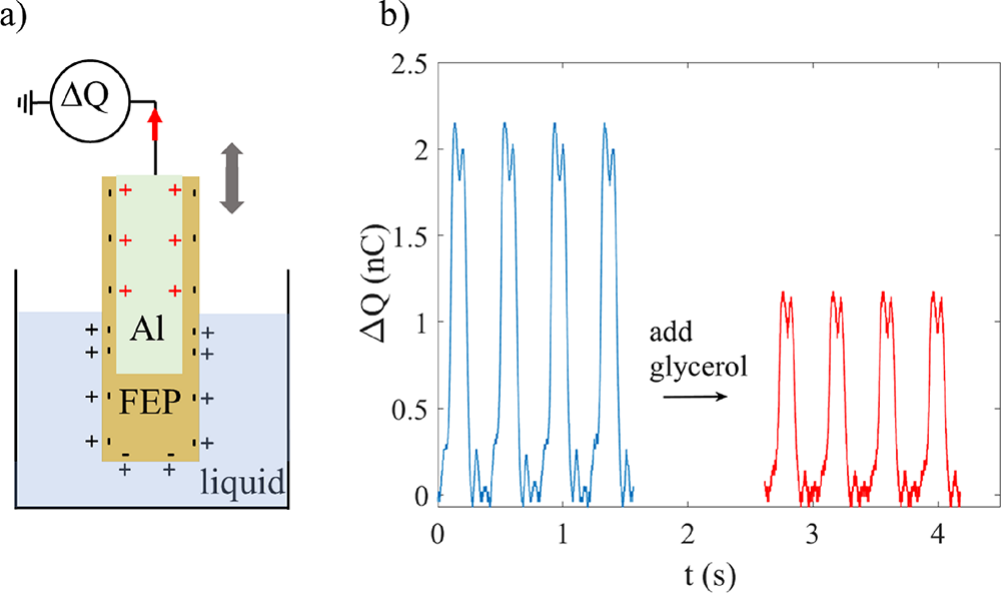
(a) Schematical drawing of the setup used to measure charge transfer. The aluminum electrode (light green) is covered by FEP polymer film (brown) and moved up and down on a cantilever. (b) The measured charge transfer decreases when one adds glycerol to water.

The experimental setup used in this study was reported in refs (27, 30) and is shown schematically in Figure 1. A 0.03 mm-thick aluminum electrode of width 22 mm and height 35 mm was glued on a polystyrene substrate and sealed by fluorinated ethylene propylene (FEP) of thickness 50 μm (Dupont). The lower-edge FEP film was 15 mm below the lower edge of the aluminum electrode. The liquid only came in contact with the FEP surface, and not the metal. A thin electrical wire was attached to the aluminum electrode and connected to a Keithley 6514 electrometer, which measures the change in charge as the FEP-covered electrode is dipped into the liquid. The liquid was held in a polystyrene beaker filled to a fixed liquid level of 70 mL. As demonstrated in refs (27, 30), the main charge transfer occurs when the edge of the covered aluminum electrode moves into water, and this occurs over a region of the order of one millimeter.

In order to obtain reliable measurements, the FEP-covered electrode was dipped repeatedly up and down in the liquid with nearly harmonic oscillations at an oscillation amplitude of 8 mm and a frequency of 2.5 Hz, corresponding to a velocity of approximately 0.1 m/s when the three-phase contact line passes the location of the edge of the metal electrode. The velocity was confirmed with ultrasonic velocity measurements using a similar setup as in ref (31). These settings were used for all the experiments reported here, and it was found that the transferred charge for deionized water was +2.0 nC to within ±0.2 nC even when waiting for several months to repeat the procedure. During all the experiments done in the current work, the temperature was kept constant at 20 ± 2 °C.

The water used was deionized and ultrapure (resistivity 18.2 MΩcm, Millipore). The glycerol was 99% pure (Sigma-Aldrich G9012—500 mL), whereas the methanol was ≥99.8% pure (Sigma-Aldrich 32213—1L). NaCl (Sigma-Aldrich, S2014—500 g) was used to control the ion concentration. All chemicals were used as received. The infrared spectra of methanol–water and glycerol–water mixtures with or without salt were recorded to monitor the interactions in the different salt-containing liquid mixtures. An Interspec 200-X FTIR spectrometer was used with an ATR crystal to record the reflectance from the solution deposited on the crystal.

In the dipping studies considered here, the glycerol–water and methanol–water fractions had to be kept low to not alter the experimental setup significantly. The largest mole fraction of glycerol in water considered in this study was *f*_m_ ≈ 0.07, corresponding to about 20 mL of glycerol in 50 mL of water. Above this fraction, the viscosity increased so much that it started perturbing the dipping mechanics, thereby making it difficult to measure the charge reliably with the technique used here. It was also found that upon increasing the methanol mole fraction beyond 0.16, there were instabilities (splashing) and bubble formation upon dipping, and it was found that the charge measurements were unreliable such that the uncertainty between independent measurements by far exceeded ±0.2 nC. For the reasons stated above, the mole fraction was limited upward to 0.16 for methanol and 0.07 for glycerol. The high vapor pressure of methanol causes it to vaporize quicker than water and to ensure that changes in the liquid level or mole fraction did not influence the results; every dipping experiment was finished within 10 min. To ensure consistency, the same procedure was implemented also for the glycerol–water mixtures.

FEP is hydrophobic, and the static contact angle is θ_s_ = 113 ± 2°, as reported in ref (14). Similarly, the advancing and receding contact angles using the tilted plate method described in refs (14, 43) were reported to be θ_f_ = 128 ± 5° and θ_r_ = 100 ± 1°, respectively. While we have found that there is some variation in the exact values with the batches of FEP obtained from the commercial vendor, the variations in contact angle do not normally exceed a few degrees. Thus, it is clear that FEP is hydrophobic during both receding and advancing of contact lines. Similar measurements using 10 μL of glycerol droplets revealed a static contact angle θ_s_ = 98 ± 6° and advancing and receding contact angles upon tilting θ_f_ = 107 ± 5° and θ_r_ = 93 ± 3°, respectively. The static contact angle is comparable with that reported in a previous report^44^ and demonstrates that also glycerol gives rise to large contact angles. An extensive study of the wetting for glycerol–water mixtures is not done here, but it is found that for a mole fraction of 0.05, the static contact angle becomes θ_s_ = 110 ± 4°, i.e., in the same range as that of pure water to within the uncertainty of the measurements. Adding salt to the mixture up to a concentration of 10 mM gives θ_s_ = 108 ± 5°, which is also within the range of water if uncertainty is accounted for. Thus, adding small amounts of glycerol or small amounts of salt does not alter the wetting properties significantly.

Measurements of a 10 μL pure methanol droplet on FEP give θ_s_ = 61 ± 7°, θ_f_ = 74 ± 3°, and θ_r_ = 50 ± 4° and therefore considerably smaller contact angles than obtained for both water and glycerol. A methanol–water mole fraction of 0.15 gives rise to a static contact angle θ_s_ = 96 ± 3°, which upon adding salt to a concentration of 10 mM becomes θ_s_ = 97 ± 2°. While adding methanol to water does change the static contact angle, even for the relatively small mole fractions considered here, the introduction of salt to this mixture does not appear to change the static contact angle further.

## Results and Discussion

### Influence of Fraction of Glycerol or Methanol in Water

**Figure 2:**
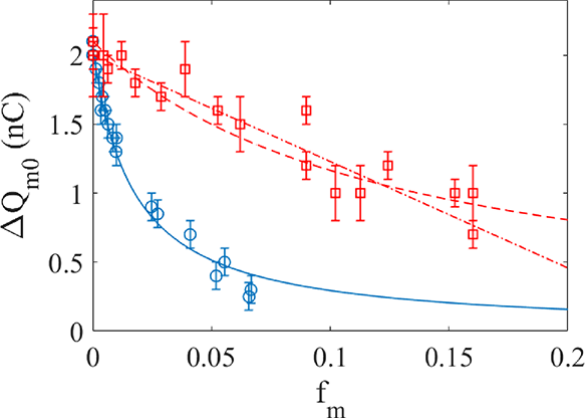
Blue circles represent the maximum charge transfer measured for different fractions of glycerol in water, and the blue line is a fit of eq 1 with *Q*_0_ = 2.1 nC and *K*_A_ = 60. The red boxes represent the maximum charge transfer measured for different fractions of methanol in water, and the red dashed line is a fit of eq 1 with *Q*_0_ = 2.1 nC and *K*_A_ = 8.0. For comparison, the red dashed–dotted line is a linear fit of *Q*_0_ – *af*_m_ with *Q*_0_ = 2.0 nC and *a* = 7.7 nC.

The charge transfer was measured for different mole fractions (*f*_m_) of methanol and glycerol in water, and the corresponding charge transfer is displayed in Figure 2 as red squares (methanol) and blue circles (glycerol).

It is seen from Figure 2 that the charge transfer decreases significantly more strongly with the glycerol fraction than with the methanol fraction. This can be modeled using a theory for the water activity, as presented in ref (29). Here, one may argue that the activity of water molecules at the solid–liquid interface is quenched by methanol or glycerol molecules. In pure water, water molecules are in principle free to participate in the hydrogen bonding network and may interact with the polymer surface by aligning their OH groups and contributing surface protons. The charge transfer in pure deionized water (*Q*_0_) was in ref (30) explained as a result of the product of surface proton activity and the equilibrium constant, stating how easy it is for the surface protons to participate in the electrical double layer. The initial charge *Q*_0_ in pure water was suggested in ref (30) to be given by *Q*_0_ ≈ *eN*_p_*K*_b_ × *a*_(H^+^)_s__, where *e* is the elemental charge (*e* = 1.6 × 10^–19^ C), *N*_p_ is the number of sites forming a negative charge on the surface (typically of the order of 10^10^–10^11^), *K*_b_ is an equilibrium constant describing the association of the hydrogenic charges with the electrical double layer, and *a*_(H^+^)_s__ is the surface activity of hydrogen ions. The theory of ref (30) does not allow one to independently extract *K*_b_ and *a*_(H^+^)_s__ from charge measurements alone, but their product was estimated to be *K*_b_*a*_(H^+^)_s__ ≈ 0.38 for the hydrophobic polymer surfaces used here.

When methanol or glycerol is introduced, some of the previously free water molecules now experience hydrogen bonding with the methanol or glycerol molecules in such a manner as to prevent the formation of charge and the subsequent transfer of charge from the electrical double layer. These bound water molecules are associated with activity *a*_b_, whereas the remaining free water molecules that can participate in the charge transfer are associated with activity *a*_f_. The sum of the activities of free and bound water, *a*_b_ + *a*_f_, is assumed to be constant. The fraction of water that can participate in charge transfer is now given by the quenching factor γ_A_ = *a*_f_/(*a*_f_ + *a*_b_). The binding between methanol or glycerol and water is further associated with an equilibrium constant, *K*_A_, such that the quenching factor is given by γ_A_ = 1/(1 + *K*_A_*f*_m_). The product of the molar fraction and the equilibrium constant is determined by the ratio of bound to free water activity, *K*_A_*f*_m_ = *a*_b_/*a*_f_. The charge transfer can then be given as
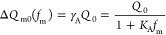
1

Fitting eq 1 to the experimental data gives *Q*_0_ = 2.1 nC and *K*_A_ = 60 for glycerol (blue line), and *Q*_0_ = 2.1 nC and *K*_A_ = 8.0 for methanol (red, dashed line). While the charge transfer of water-glycerol mixtures is relatively well described by eq 1, it is not so obvious that the data for methanol are best described by eq 1 due to the large fluctuations. For comparison, a best linear fit of *Q*_0_ – *af*_m_ with *Q*_0_ = 2.0 nC and *a* = 7.7 nC is shown as a dash–dotted line in Figure 2. As methanol is highly volatile and has smaller viscosity than water, it could be that the large uncertainties are due to altered hydrodynamics with splashing and microscopic bubble formation, although this was not directly visually observable in the experiments with low mole fractions.

**Figure 3:**
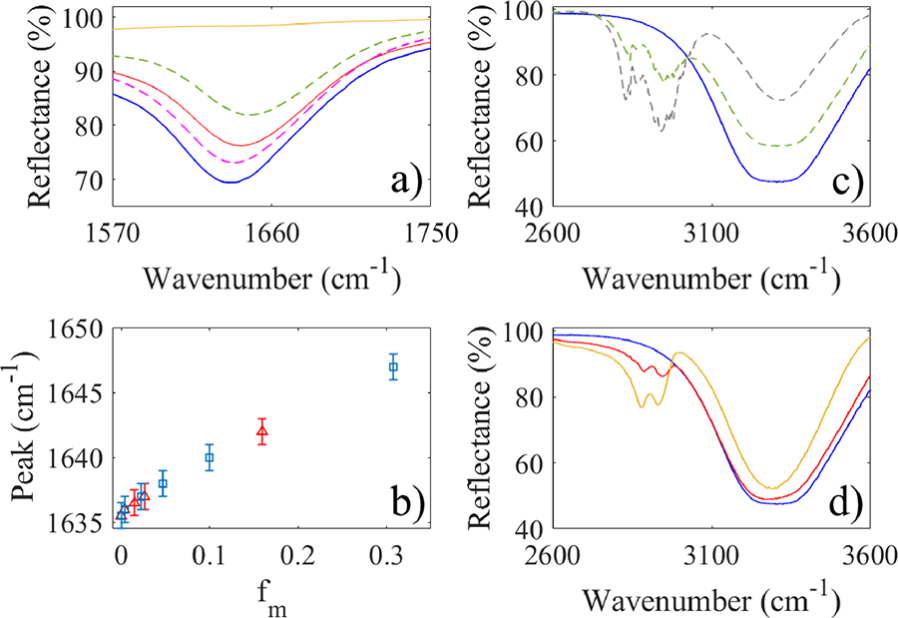
Infrared spectra of different pure liquids and mixtures. In (a), the orange solid line corresponds to glycerol, the dashed magenta line a water–methanol mixture with *f*_m_ = 0.0471, the solid red line a water–glycerol mixture with *f*_m_ = 0.1595, and the dashed green line a water–methanol mixture with *f*_m_ = 0.3077. In (b), the wavenumber at the peak of the HOH bend in (a) is shown as a function of the mole fraction of glycerol (red triangles) and methanol (blue squares). In (c), the dashed green line represents a water–methanol mixture with *f*_m_ = 0.3077 whereas the dashed gray line corresponds to pure methanol. In (d), the orange solid line corresponds to glycerol while the solid red line a water–glycerol mixture with *f*_m_ = 0.1595. In (a–d), the solid blue line represents the infrared spectrum of water.

From Figure 2, it is clear that the detailed interaction between methanol or glycerol and water has a considerable impact on the charge transfer quenching equilibrium factor *K*_A_. One may question whether this change is somehow associated with bond characteristics, for which infrared spectroscopy may provide further insight. A well-known characteristic peak in the water infrared spectrum occurs near 1640 cm^–1^ (see Figure 3a), a region in which both pure methanol and pure glycerol appear featureless. The absorption peak near 1640 cm^–1^ is attributed to HOH bending and is also known in the literature to blue-shift with increasing glycerol fraction in water^45−47^ or ethanol–water fraction.^48^ Such a blue shift is also found in our investigations (see Figure 3b). However, we also find that introducing methanol into water gives rise to a rather similar change in the peak absorption frequency versus mole fraction as for glycerol–water mixtures, as seen in (Figure 3b). Therefore, both the methanol and glycerol molecules alter the water structure in such a manner that the HOH-bending vibrations become faster and this has previously been interpreted somewhat loosely to be due to an increase in hydrogen bond activity among water molecules.^45^ A recent study on glycerol–water mixtures combining infrared spectroscopic with dielectric spectroscopy at lower frequencies suggested that water molecules contributing to hydrogen bonds in the hydration layer near glycerol molecules lose intermolecular bending coupling.^47^

As seen in Figure 3c,d, pure methanol and glycerol exhibit an asymmetric CH_2_ stretch near 2930 cm^–1^ and a symmetric CH_2_ stretch at slightly lower wavenumbers (2830–2880 cm^–1^). Both of these peaks are blue-shifted as more water is mixed with either methanol or glycerol, which is believed to be due to the formation of a stronger hydrogen bonding network of OH groups as more water is mixed in, thus leading to more strained CH_2_ groups, which vibrate at higher frequencies.

The OH-stretching band of water in the region 3200–3400 cm^–1^ is relatively broad and is composed of several components.^44−46^ For pure methanol or glycerol, the peak is narrower as seen in Figure 3c,d. Thus, there is a gradual transition from the infrared spectrum of water to that of methanol or glycerol. However, proper interpretation of the combined spectra requires careful removal of optical effects (refractive index, polarization of radiation, etc.) and combination with other techniques (e.g., dielectric spectroscopy). A recent study combining infrared spectroscopy with other techniques has demonstrated that careful interpretation may reveal some details of the hydrogen bond configuration when glycerol is introduced,^47^ but this is outside the scope of the current work.

From the data in Figure 3, we observe that methanol and glycerol influence the HOH bending in a rather similar manner and it appears that neither the changes in CH_2_ stretch or the OH bend can explain that the charge transfer quenching equilibrium factor *K*_A_ is considerably larger for glycerol than for methanol. It should be mentioned that ATR-FTIR is a method wherein infrared light penetrates a few micrometers into the liquid, which means that both surface and bulk contributions are observed. More importantly, it is possible that spatial heterogeneity on a nanometer scale, which cannot be resolved by any infrared spectroscopy technique, contributes to observed charge transfer. A possible way to interpret the difference in *K*_A_ is to assume that there are fewer water molecules bound to methanol than to glycerol in the hydrogen bond network. Methanol molecules are small, with a methyl group orienting away when hydrogen bond networks are formed. At low mole fractions, methanol molecules in the bulk are believed to form extended structures in the form of short chains or percolating networks within the liquid.^49−51^ One could imagine that these structures do not interfere with water activity at the fluoropolymer surface, thus allowing a relatively high surface hydrogen ion activity, which might be responsible for the small *K*_A_ in methanol. On the contrary, the larger glycerol molecules have three hydroxyl groups and may more effectively bind up water near the surface, thus preventing charge transfer.

### Influence of Salt Concentration

**Figure 4:**
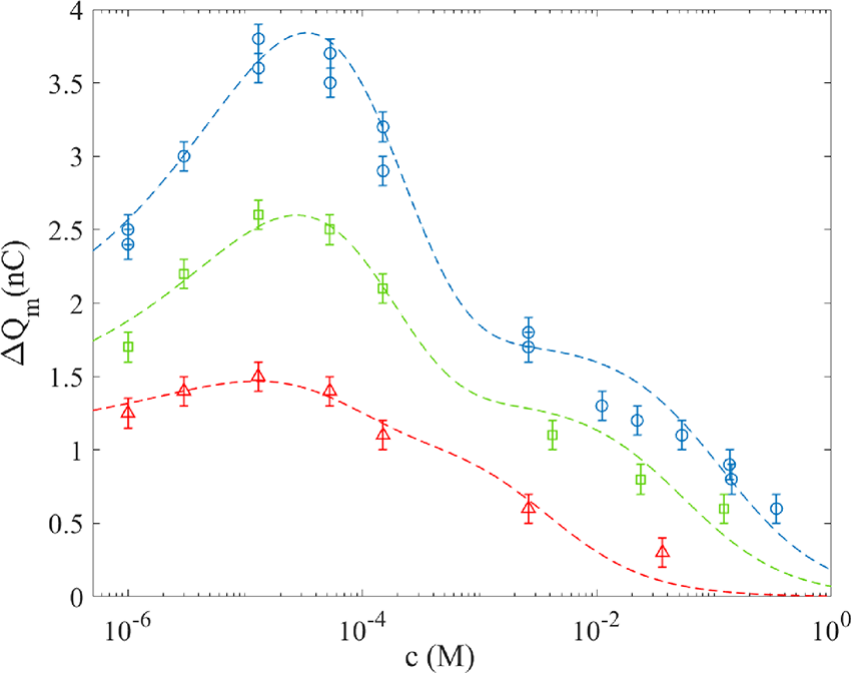
The measured charge as a function of NaCl concentration for the methanol-to-water fraction *f*_m_ = 0 (blue circles), *f*_m_ = 0.069 (green boxes), and *f*_m_ = 0.15 (red triangles). The dashed lines are fits to eq 3, with the parameters given in the text.

The charge transfer was measured as a function of ion concentration (*c*) in the presence of pure water and different mole fractions of a methanol. The results are presented in Figure 4. While the blue circles correspond to NaCl mixed into pure water (*f*_m_ = 0), the green squares correspond to NaCl mixed into a methanol/water mole fraction *f*_m_ = 0.069 and the red triangles to *f*_m_ = 0.15. From the data in Figure 4, one can observe that the charge transfer increases with added amount of NaCl as long as the ion concentration is small and then reaches a maximum before decreasing with large concentrations. For all of the measured methanol–water fractions, there is a maximum charge transfer occurring at salt concentrations of the order of 10^–5^ M.

A simple theory accounting for both quenching and removal of ions from the electrical double layer was presented in ref (30). In water containing glycerol or methanol, the charge Δ*Q*_m0_ that can be transferred is governed by the activity of free water and therefore the surface protons caused by the alignment of hydroxyl groups in the hydrogen bond network near the polymer surface. When salt is added, more ions are contributed to the electrical double layer. The Debye–Hűckel theory is assumed to be valid for the low salt concentrations using here, as supported also for other aqueous systems of low concentrations,^52^ such that the charge density ρ can be approximated as .^53^ Here, ε_0_ is the permittivity of vacuum, ε = 80 the relative permittivity of water, Φ is the electric potential, and *x* is the distance away from the polymer surface. *B* (*B* < 0) is considered a constant depending on the surface potential, as discussed in refs (27, 53). The inverse Debye length can for the temperature considered here be given in terms of the salt concentration *c* (moles per liter) as  (nm^–1^).^53^ As the three-phase contact line passes the polymer surface, a net positive charge Δ*Q* due to the added ions is removed from the electrical double layer. We attempt to calculate this additional net positive charge caused by adding salt by assuming that the ions in the electrical double layer further from the polymer surface than a shear distance *x*_s_ are removed by fluid flow, such that
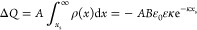
2where *A* = *wL*, with *L* the effective charge collection length^27,30^ and *w* the horizontal width of the metal electrode. If one assumes that *x*_s_ remains constant, eq 2 states that the charge removed from the electrical double layer increases as  for small concentrations and then reaches a maximum at *c*_max_ = 1/(3.3*x*_s_)^2^, before falling off as  at larger concentrations. However, this exponential falloff at higher concentrations is too strong to explain the observed decay of charge transfer with concentration, and we will argue that this decay could better be explained by quenching due to reduced water activity as described in ref (29).

So far, we have argued that the methanol or glycerol molecules alter the hydrogen bonding network near the polymer surface, thus reducing the surface proton activity and reducing how easy it is for the surface protons to participate in the electrical double layer. This gives rise to the quenching constant γ_A_ and the charge given in eq 1 in absence of added salt. The ions introduced by adding salt alter the electrical double layer to provide an additional contribution to the charge (as given by eq 2). However, some of the cations (Na^+^) may also penetrate even further and alter the surface proton activity to reduce the net negative charge near the surface of the hydrophobic polymer. This process may take longer time than the formation of the hydrogen bonding network or the formation of the outer electrical double layer since these cations need to move past the entire electrical double layer and also possibly disrupt the hydrogen bonding network. In such a scenario, the ions therefore quench both the charge due to the glycerol–water or methanol–water network as well as the charge that can be removed from the electrical double layer up to a shear distance *x*_s_. This results in a global quenching factor γ_p_, which applies to both Δ*Q* and Δ*Q*_m0_.

The quenching factor γ_p_ can be estimated by considering a fraction of free water associated with an activity *a*_f_* and an ion-bound activity *a*_b_*, where the sum of *a*_f_* + *a*_b_* is constant. We assume that these activities are not correlated with *a*_f_ and *a*_b_ in the previous section since the mixtures are dilute. The quenching factor is given by γ_p_ = *a*_f_*/(*a*_f_* + *a*_b_*) = 1/(1 + *K*_qp_*c*), where *K*_qp_ = *a*_b_*/*ca*_f_* is an equilibrium constant. The total charge transfer induced in the metal electrodes can then be expressed as
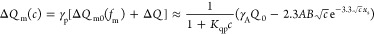
3

Using the function nlinfit in MATLAB to fit eq 3 to the experimental data for salt in pure water, i.e., the blue circles in Figure 4, gives γ_A_*Q*_0_ = 1.7 × 10^–9^ C, *AB* = −4.3 × 10^–7^ Vm^2^, *K*_qp_ = 8.5 M^–1^, and *x*_s_ = 52 × 10^–9^ m. The width of the metal electrode is *w* = 1.0 10^–2^ m, thus giving *L* = *A*/*w* = 4 × 10^–4^ m if one assumes *B* = −0.1 V. Thus, the charge is collected within roughly 0.4 mm near the metal edge, in reasonable agreement with previous simulations.^27^ The value *K*_qp_ = 8.5 M^–1^ for the equilibrium quenching constant found here is smaller than the value *K*_qp_ = 20 M^–1^, reported in ref (30) but still within the same order of magnitude. The reason for this is mainly that the fit presented here has been optimized using the function nlinfit to the particular data set in Figure 4, while in ref (30), the fit was made to a larger set of data comprising a range of different salts. With this in mind, the value *x*_s_ = 52 × 10^–9^ m found in Figure 4 compares reasonably well with the value *x*_s_ = 60 × 10^–9^ m found in ref (30).

Equation 3 was also fit to the experimental data in Figure 4 for *f*_m_ = 0.069 (green boxes in Figure 4), giving γ_A_*Q*_0_ = 1.3 × 10^–9^ C, *AB* = −2.8 × 10^–7^ Vm^2^, *K*_qp_ = 18 M^–1^, and *x*_s_ = 58 × 10^–9^ m. Similarly, for *f*_m_ = 0.15 (red triangles in Figure 4), one obtains γ_A_*Q*_0_ = 1.1 × 10^–9^ C, *AB* = −1.2 × 10^–7^ Vm^2^, *K*_qp_ = 269 M^–1^, and *x*_s_ = 83 × 10^–9^ m. It can therefore be observed that while the value for the shear distance *x*_s_ does not change very much, the equilibrium constant *K*_qp_ increases significantly with an increasing methanol fraction in water.

**Figure 5:**
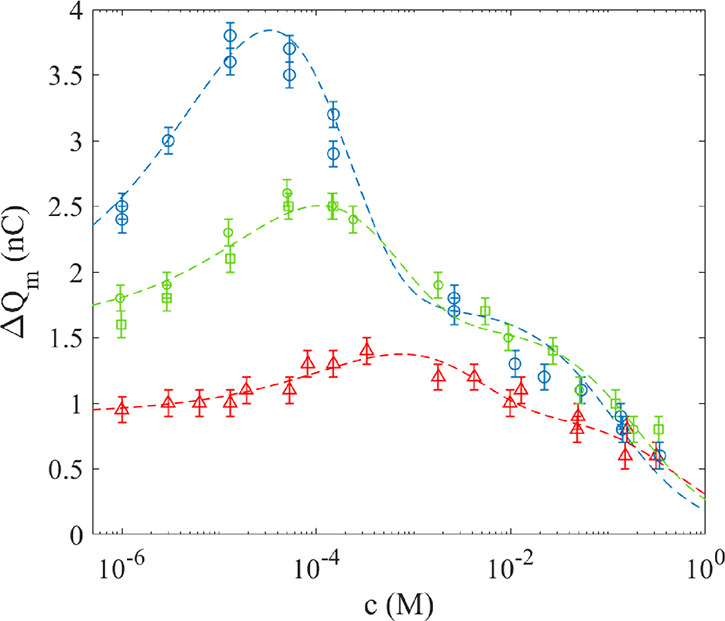
The measured charge as a function of NaCl concentration for glycerol to water fraction *f*_m_ = 0 (blue circles), *f*_m_ = 0.0035 (green boxes), and *f*_m_ = 0.027 (red triangles). The dashed lines are fit of eq 3 to the experimental data with parameters given in the text.

The charge transfer measured as a function of the ion concentration for different volume fractions of glycerol in water is presented in Figure 5. For reference, the case of adding NaCl into deionized water is displayed in Figure 5 as blue circles, while green squares correspond to NaCl mixed into a glycerol/water volume fraction *f*_m_ = 0.0035 and the red triangles correspond to *f*_m_ = 0.027. For all data sets measured, the charge transfer increases with added NaCl for small concentrations before reaching a maximum, after which the charge transfer monotonously decreases. It is found that also all glycerol–water mixtures exhibit a maximum charge transfer at a given salt concentration. Interestingly, the maximum of the charge transfer occurs at about 10^–5^ M for pure water but increases to about 4 × 10^–4^ M for a glycerol/water concentration of *f*_m_ = 0.027. Fitting eq 3 to the experimental data gives the results listed in Table 1, which also shows the fitting parameters for methanol–water mixtures, such as those of Figure 4.

**Table 1:** Fitting Parameters Obtained when Fitting eq 3 to the Experimental Data in Series Such as Those of Figures 4 and 5 Plus Additional Experiments^a^

mole fraction	quenching due to methanol/glycerol	quenching due to added ions	surface potential area	shear distance	liquid mixture
*f*_m_	γ_A_*Q*_0_ (nC)	*K*_qp_ (M^–1^)	*AB* (nVm^2^)	*x*_s_ (nm)	
0	1.73	8.5	–431	52	water
0.0054	1.75	5.3	–205	36	glycerol–water mixture
0.0141	1.54	3.4	–80	19	
0.0158	1.64	7.1	–103	29	
0.0345	1.25	3.3	–28	11	
0.0274	0.91	3.3	–18	12	
0.0274	0.94	1.5	–21	11	
0.0308	1.52	8.9	–235	49	methanol–water mixture
0.0690	1.34	18	–283	58	
0.1509	1.11	269	–117	83	

a The uppermost row provides a simplified description of the physical meaning associated with the parameters.

Table 1 also shows the results of additional experiments done at the same or different mole fractions of methanol or glycerol in water. Notably, both *x*_s_ and *K*_qp_ decrease strongly with the glycerol molar fraction.

In both Figures 4 and 5, one notices that the theoretical curves exhibit two different decay modes, most clearly seen in the region 1–100 mM. The reason for this is that eq 3 is a multiplication of two expressions. The contributed charge Δ*Q* contains an expression falling off as , which means that the contribution of added ions (due to NaCl) in the electrical double layer falls of quickly at larger concentrations since the electrical double layer contracts within the shear distance *x*_s_. When the contribution of added ions is negligible, the main charge transfer decay mechanism is due to quenching by the term 1/(1+*K*_qp_*c*), which falls more slowly. The exact location of the shift in decay caused by electrical double-layer contraction to quenching depends on the fitted values of *x*_s_ and *K*_qp_. There are indications in some of the experimental data that there may be two different decay modes, but from the available data, one cannot state with confidence whether these decay modes are those of the theoretical predictions of eq 3. It could therefore also be that a more comprehensive model is needed to fully account for the observed decay in charge transfer with salt concentration.

**Figure 6:**
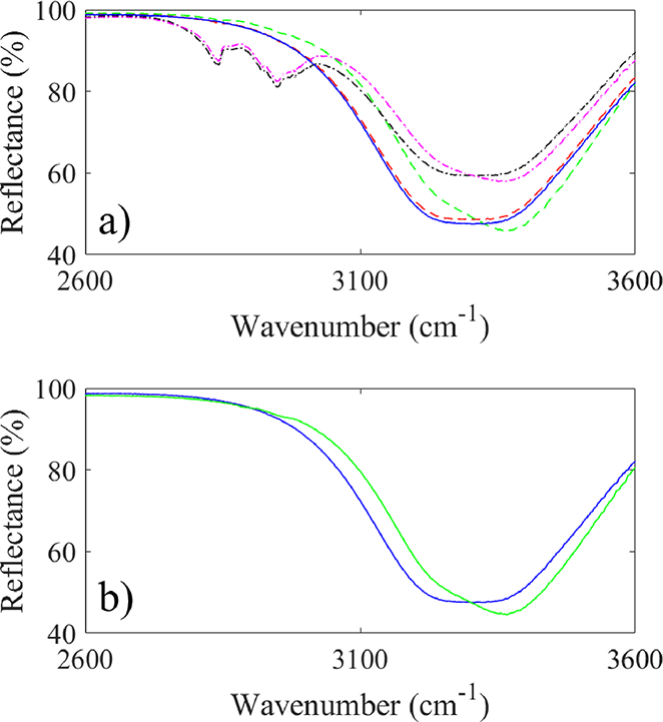
Infrared spectra of water–methanol (a) and glycerol–water (b) mixtures in the presence of salt. In (a), the dashed red line corresponds to a water–methanol mixture with *f*_m_ = 0.023 and *c* = 10 μM, whereas the green dashed line corresponds to the same mole fraction but now with *c* = 4 M. The dash–dotted black line corresponds to a water–methanol mixture with *f*_m_ = 0.308 and *c* = 5 μM, whereas the magenta dash–dotted line corresponds to the same mole fraction but now with *c* = 2 M. In (b), the green solid line represents the infrared spectrum of a glycerol–water mixture with *f*_m_ = 0.0156 and *c* = 4 M. The solid blue lines in (a) and (b) represent the infrared spectrum of water.

Adding salt to either pure water, methanol–water, or glycerol–water did not alter the CH_2_ stretching in the region 2800–2950 cm^–1^ or the HOH bending near 1640 cm^–1^ noticeably in a manner that could be detected using the available ATR-FTIR instrument. The OH-stretching band of water in the region of 3200–3400 cm^–1^ is strongly influenced at higher salt concentrations, as observed in Figure 6. This observation is consistent with those in previous studies of salt-solvated water spectra,^54−56^ wherein a similar behavior is also reported for other ions. The ions deform the O–H bonds of the nearby hydration layer and rearrange the local hydrogen bonding network in such a way to favor vibrations at higher wavenumbers. In a previous study of salt-water solution, the stretching of the OH bond was found to occur mainly linearly with salt concentrations up to about 2 M.^55^ In ref (56), it was argued that ion shielding prevents significant distortion of the OH bonds as long as the ion concentration is low. This is further confirmed in the current study for various methanol–water and glycerol–water fractions, where a similar behavior occurs in salty solutions of both glycerol and methanol with water. This may suggest that it is not changes in the OH bonds as measured by ATR-FTIR that are responsible for the shift in charge transfer with increasing glycerol–water mixture seen in Figure 5.

For these reasons, we will in the following attempt to analyze the charge transfer data in more detail by extracting parameters that are directly influenced by the environment.

### Extracted Parameters

**Figure 7:**
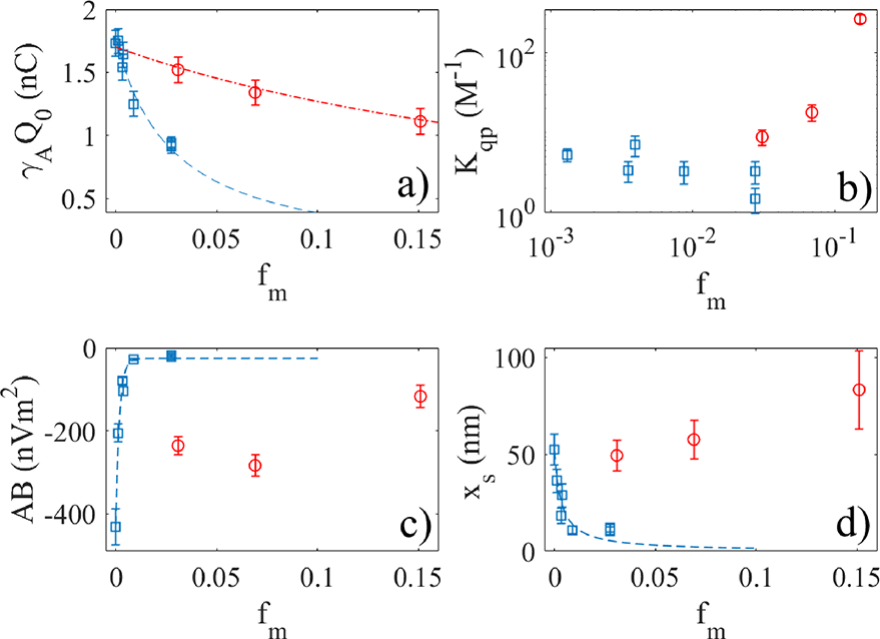
Graphs show the extracted parameters obtained from eq 3 displayed as a function of the glycerol-to-water or methanol-to-water fraction *f*_m_. (a) The quenching due to methanol and glycerol. (b) The quenching due to added ions. (c) The surface potential area. (d) The shear distance. The blue squares represent glycerol–water mixtures, and the red circles represent methanol–water mixtures. The dashed and dotted lines are fits to the extracted parameters. See text for more information.

Several more curves for different glycerol–water and methanol–water fractions were measured in addition to the experimental data shown in Figures 4 and 5, and the corresponding parameters were obtained using eq 3 presented in Table 1 and plotted in Figure 7. In Figure 7a, it is seen that γ_A_*Q*_0_ decreases monotonously with the molar fraction, as one may expect when observing Figure 2. If one assumes a function *Q*_0_/(1 + *K*_t_*f*_m_), it is found that *Q*_0_ = 1.8 nC and *K*_t_ = 36 provide a good fit for glycerol while *Q*_0_ = 1.7 nC and *K*_t_ = 3.4 for methanol. The obtained values for *K*_t_ from the data in Figure 7 are about half of the values of *K*_A_ reported for the data in Figure 2. This could be interpreted as a significant reduction in the quenching constant *K*_A_ due to methanol or glycerol molecules when salt is added. The added ions may surround the methanol and glycerol molecules and reduce their ability to quench the charge transfer. However, one must also note that the simple theory of eq 3 does not provide a perfect fit to the experimental data and that the values for *Q*_0_ are 0.3–0.4 nC smaller than the values found in Figure 2. Thus, the obtained constants *K*_t_ and *Q*_0_ are also a balance of the values for γ_A_*Q*_0_ and *AB* must be made to make a fit to the entire data sets such as those of Figures 4 and 5. Nonetheless, it appears that *Q*_0_/(1 + *K*_t_*f*_m_) describes the data sets of γ_A_*Q*_0_ reasonably well as seen in Figure 7a, thus providing further confidence in the quenching model adopted from ref (29).

In a very recent study of sucrose-induced reduction of charge transfer as water droplets slide along an FEP surface between electrodes, it was argued that sucrose has an effect on the hydrogen bonding to promote water ionization and produce hydrogen ions, which interact directly with the charges at the solid surface.^57^ It was demonstrated experimentally in ref (30) that direct introduction of hydrogen ions using different types of acids does indeed give rise to a reduction in charge transfer, which was attributed to hydrated protons moving efficiently through the hydrogen bond network with water as a catalyzer only, thus allowing them to interact directly with the solid surface charge states. Methanol and glycerol do not give rise to free protons and interact with water through hydrogen bonding, and their mixtures with water are therefore well described by the quenching mechanism in eq 1. If sucrose also alters the hydrogen bonding network in a similar manner without producing hydrogen ions that directly interact with the surface, it can perhaps explain why the charge transfer presented in Figure 3 h in ref (57) shows a similar behavior to that seen in Figure 3 for methanol–water and glycerol–water mixtures in the current work. If surface active hydrogen ions are produced in the sucrose–water mixtures of ref (57), one might expect to see a different behavior closer to that reported in Figure 4 in ref (30).

The equilibrium constant *K*_qp_ reduces from 8.5 to 1.5 M^–1^ as the glycerol–water fraction increases from zero to 0.027. On the other hand, K_qp_ increases from 8.5 M^–1^ to 269 M^–1^ when the methanol–water fraction increases from zero to 0.15. By comparison of Figures 4 and 5, it is seen that the relative quenching is significantly larger in methanol–water than in glycerol–water for larger salt concentrations. This difference in behavior might be due to glycerol molecules prohibiting quenching γ_p_ of charge transfer by salt ions more efficiently than methanol at higher concentrations, which can be understood as the three hydroxyl groups of glycerol altering the hydrogen bonding network to hinder the cations (Na^+^) that otherwise would be moving toward the polymer surface to reduce surface proton activity. It is known that introduction of methanol into water reduces the dielectric permittivity and gives rise to ion pairing.^58^ While the exact mechanism remains unclear, one may speculate whether the formation of extended structures of methanol in water as described in refs (49−51) causes enhancement of ion concentration in the water-rich domains and near the surface such that the charge transfer is more strongly reduced than expected for homogeneous mixtures at large methanol fractions.

When fitting eq 2 to data such as in Figures 4 and 5, it was found that the factor *AB* also changed with the glycerol–water volume factor, as shown in Figure 7c. The blue squares are the extracted values for glycerol–water mixtures, whereas the red circles represent methanol–water mixtures. Note that the parameter AB obtained for glycerol–water mixtures decreases monotonously with mole fraction. As a guide for the eyes, the blue dashed line in Figure 7c represents a fit of the function *y* = *y*_0_ + *y*_1_exp(−*af*_m_) to the experimental data, where *y*_0_ =–25.6 nV/m^2^, *y*_1_ = −400.3 nV/m^2^ and *a* = 546. For methanol–water mixtures, one also notes a decrease in |*AB*| with the mole fraction, but the trend is not clear.

The theory behind eq 3 may be used to partially explain these observations for the change in AB with *f*_m_, if one first assumes that the effective area *A* = *wL* over which charge is collected in the vicinity of the metal electrode edge is constant. Under such circumstances, only parameter *B* in *AB* changes with *f*_m_ and one could interpret this as less charge is removed from the electrical double layer with increasing mole fraction. To see this, note that in the classical Gouy–Chapman theory, one has  sufficiently far away from the Stern layer,^27,53^ where *e* is the electronic charge, *k*_B_ is Boltzmann’s constant, *T* is the temperature, and ϕ_d_ is the potential associated with the innermost part of the diffusive electrical double layer where the Stern layer begins. When , it is seen that , whereas for , one has *B* ≈ – ϕ_d_. According to Figure 7c, it is seen that for *f*_m_ = 0, one has *AB* ≈ −4 × 10^–7^ Vm^2^, which gives *B* ≈ −100 mV if *w* = 1 × 10^–2^ m and *L* = *A*/*w* = 4 × 10^–4^ m. For a water–glycerol fraction *f*_m_ = 0.027, one finds *AB* ≈ −2 × 10^–8^ Vm^2^, which gives *B* ≈ −5 mV if *w* = 1 × 10^–2^ m and *L* = 4 × 10^–4^ m. In this interpretation, the surface potential has been reduced significantly by the introduction of glycerol. For methanol, the reduction in surface potential is much smaller even for higher mole fractions, since the product *AB* has a larger absolute value, as seen from Figure 7c. This is consistent with the observation in Figure 2 that methanol has less impact on charge transfer at small mole fractions.

When interpreting the values of the surface potential in the manner suggested above, one must assume a simple relationship between *B* and ϕ_d_ and that the value for *A* remains constant. These assumptions need further verification, but this is outside the scope of the current work. However, the claim that glycerol has a much stronger impact on surface potential than methanol might be further verified from the extracted data in Figure 7d, where it is seen that *x*_s_ decreases monotonously for increasing the glycerol–water fraction but remains unaltered or even increases with increasing water–methanol fraction. The value of *x*_s_ is connected to the ion concentration, which gives the maximum charge transfer, and this does not change dramatically (from 52 to 83 nm) as the methanol–water fraction increases. On the other hand, increasing the glycerol–water from 0 to 0.027, decreases x_s_ from approximately 52 to 11 nm in something that appears to be a well-defined manner.

**Figure 8:**
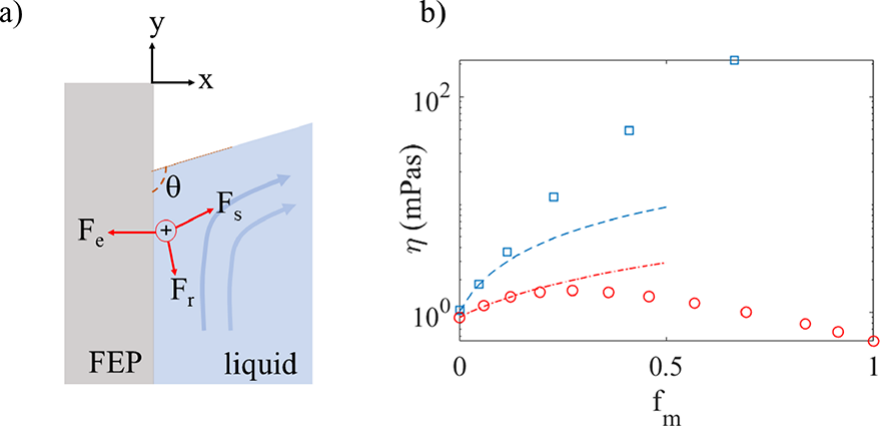
(a) The forces acting on a single ion; see text for details. (b) Graph showing the dynamic viscosity of glycerol–water (blue boxes) and methanol–water (red circles) for different mole fractions. The data for glycerol are extracted from ref (59), while the data for methanol are extracted from ref (60).

As observed in Figure 7d, *x*_s_ decreases with the glycerol–water fraction for small *f*_m_ but appears to saturate at a lower value when the fraction gets bigger. This can be explained by noting that the removal of ions from the electrical double layer is a competition between the viscous shear forces trying to remove the ions and the electrical forces holding them back. A schematic drawing of the forces is depicted in Figure 8a. The electrical attraction force on the positive ion a distance *x*_s_ away from the FEP surface charge is approximately given by *F*_e_ ≈ σ*E* ≈ σ*V*_0_(*f*_m_)/*x*_s_ and is assumed to act perpendicular to the surface. Here, *V*_0_(*f*_m_) is the potential difference over the distance *x*_s_ and σ is the effective surface charge density. Both of these parameters may in principle depend on the mole fraction since glycerol and methanol molecules quench the activity of the ions involved in charge transfer. Here, we will for simplicity of discussion lump this dependency into *V*_0_(*f*_m_) and assume that σ is constant. Furthermore, a force *F*_s_ due to the liquid flow and a reaction force *F*_r_ due to the proximity of the liquid–gas interface also act on the ion. From Figure 8a, it is seen that *F*_e_ = *F*_r,*x*_ + *F*_s,*x*_, where *F*_s,*x*_ is the *x*-component of the fluid flow force caused by the viscous liquid and *F*_r,*x*_(*f*_m_) is the *x*-component of the resisting force due to interactions with the solid–liquid and liquid–gas interfaces. Note that inertia is neglected due to the very small mass of the ion. The fluid flow force *F*_s_ depends on the direction of the flow near the contact line and has both *x*- and *y*-components depending on the position of the ion within the flow near the contact line. The direction of the resisting force *F*_r_ is hypothesized to be perpendicular to the air–liquid interface, but that depends on the position of the ion and the detailed interaction near the contact line. Here, the purpose is only to make a simple model to allow further discussion of the physical mechanisms involved, with an aim to further aid an explanation of the observations in Figure 7d. We therefore make the hypothesis that the fluid flow force is given by *F*_s,*x*_ ≈ *A*η_s_(*f*_m_)*r*, where *A* is an effective area, η_s_(*f*_m_) is the surface dynamic viscosity, and *r* is the shear rate. Balancing these force components gives
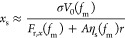
4

Here, all the parameters *V*_0_(*f*_m_), η_s_(*f*_m_), and *F*_r,*x*_(*f*_m_) may depend on the mole fraction *f*_m_ and we will therefore discuss them one by one in order to land at a possible understanding of the behavior seen in Figure 7d. The dashed line in Figure 7d shows a fit of the form *x*_0_/(1 + *z*_1_*f*_m_) to the experimental data for glycerol–water mixtures, where *x*_0_ = 52 nm and *z*_1_ = 303. Thus, eq 4 may apparently provide an explanation for the data for *x*_s_ in Figure 7d, but care must be taken.

We note that the reaction force *F*_r,*x*_(*f*_m_) is influenced by the liquid–air interface and may therefore depend on the surface tension. The surface tension of water is 73 mN/m, glycerol 64 mN/m, and methanol 23 mN/m. While water and glycerol have rather similar surface tensions, methanol exhibits significantly lower surface tension. Reducing the surface tension by adding methanol to water also reduces *F*_r,*x*_(*f*_m_).

Using the data for glycerol from ref (59) seen in Figure 8b, it is found that for small fractions (*f*_m_ < 0.1), the viscosity changes approximately according to η ≈ η_0_ + η_0_*f*_m_, where η_0_ = 1 mPas and η_1_ ≈ 17 mPas. See the blue dashed line in Figure 8b. For the methanol data extracted from ref (60), it is found that the viscosity of methanol–water mixtures can be approximated by η ≈ η_0_ + η_0_*f*_m_, where η_0_ = 0.9 mPas, η_1_ ≈ 4 mPas, and *f*_m_ < 0.2. See the red dash–dotted line in Figure 8b.

If one assumes that the viscosity experienced by ions near the FEP surface is η_s_ ≈ η, the data in Figure 8b suggest that η_s_ increases by at most a factor of approximately two in any of the experiments reported in this study. If one assumes that *F*_r,*x*_(*f*_m_) is constant and larger than zero, the corresponding change in *x*_s_ is likely to be smaller than a factor of 2 according to eq 4. By comparison with the data in Figure 7d, it appears that bulk dynamic viscosity of the mixtures alone cannot explain the difference in extracted values for *x*_s_ for methanol and glycerol unless also *F*_r,*x*_(*f*_m_) changes strongly. For glycerol–water mixtures, one does not expect *F*_r,*x*_(*f*_m_) to change strongly with *f*_m_, and it is unlikely that this factor can explain the change in *x*_s_ observed in Figure 7d. In methanol–water mixtures, *F*_r,x_(*f*_m_) may decrease with surface tension as more methanol is mixed into water, causing the static contact angle to decrease slightly, such that *x*_s_ stays constant or even increases with *f*_m_, as observed in Figure 7d.

In addition to viscosity and surface tension, the potential *V*_0_(*f*_m_) may account for the changes in *x*_s_ with changes in the methanol/water or glycerol–water fraction. As discussed above in connection with Figure 7 c, the reduction in the value of the surface potential might be larger for glycerol–water than methanol–water mixtures and this may also influence the large change in *x*_s_ with *f*_m_ seen in Figure 7d. Based on the discussion above, a possible interpretation is that the increase in viscosity and decrease in potential act together to reduce the values of *x*_s_ for glycerol–water mixtures in Figure 7d, such that a function of the form *x*_0_/(1 + *z*_1_*f*_m_) explains the data rather well. For methanol–water mixtures, the surface potential does not change very quickly as *f*_m_ increases since the quenching is comparably weaker than for glycerol–water mixtures, as seen in Figure 2. As *f*_m_ increases, the surface tension decreases while the viscosity increases for the fractions investigated, and these factors appear to balance each other such that *x*_s_ remains nearly constant or even increases by a small amount with increasing *f*_m_. At this point, it should be noted that for larger methanol–water fractions, the curves in Figure 4 broaden and the peak becomes less pronounced while the maximum charge transfer remains at the same ion concentration. The best nonlinear fit is obtained by fitting eq 3 to the experimental data. However, the curve shape provided by the simple theory of eq 3 makes it necessary to caution about possible systematic errors in *x*_s_, thus making it hard to confidently state whether this parameter remains constant or increases slightly.

## Conclusions

In the current study, it is demonstrated that both methanol and glycerol quench charge transfer as an aqueous solution moves over a hydrophobic fluoropolymer. The charge transfer quenching is stronger for glycerol than for methanol, which may be related to the formation of extended methanol structures in water that do not interfere with charge sites in the same manner as for glycerol. It is demonstrated that in the case of glycerol/water, the salt concentration giving rise to maximum charge transfer is controlled by the glycerol mole fraction, a feature not found for methanol–water mixtures. A model is presented to explain these unexpected results. Parameters extracted from the model suggest that reduction in ion shear distance in the electrical double causing the glycerol-induced shift in charge transfer is due to a combination of reduced electrical surface potential and increased surface viscosity as the glycerol mole fraction increases. These findings may help one further understand how to control charge transfer when different liquids come in contact with fluoropolymers, which is required to optimize sensors and energy harvesting systems based on this phenomenon.
